# The estimated effect of graphic warning labels on smoker’s intention to quit in Shanghai, China: a cross-sectional study

**DOI:** 10.1186/s12889-021-12257-8

**Published:** 2021-11-26

**Authors:** Ruiping Wang, Yan Qiang, Yan Zhu, Xiangjin Gao, Qiong Yang, Bin Li

**Affiliations:** 1 Clinical Research & Innovation Transformation Center, Shanghai Skin Diseases Hospital, Tongji University, 1278 Baode Road, Jing’an District, Shanghai, 200443 China; 2 School of Public Health, Shanghai University of Traditional Chinese Medicine, Shanghai, China; 3 Office of Clinic management, Shanghai Skin Diseases Hospital, Tongji University, Shanghai, China; 4 Songjiang Maternal and Children Healthcare Hospital, Shanghai, China; 5 Songjiang Fang Song Community Health Service Center, Shanghai, China

**Keywords:** Graphic warning label, Cigarette package, Smoking duration, Smoking intensity, Current smoker, Smoking cessation intention

## Abstract

**Background:**

Tobacco consumption is the leading cause of death worldwide. Overwhelming studies demonstrate graphic warning labels (GWLs) on cigarette packs are effective in eliciting negative response to tobacco smoking, modifying beliefs about tobacco dangers, and increasing reported intention to quit, but the estimated effect of GWLs on smoking cessation intention among smokers is still limited in China. In this study, we aim to understand the smoking intensity, smoking duration and smoking cessation intention among current smokers, and to explore how their smoking cessation intention would be influenced by the GWLs in Shanghai.

**Methods:**

From January to June 2021, we totally recruited 1104 current smokers in Songjiang district and Fengxian district of Shanghai by multistage sampling design. We used Android pad assisted electronic questionnaire for data collection, and then implemented logistic regression for odds ratio (OR) and 95% confidence interval (CI) calculation to explore how smoking cessation intention would be influenced by the GWLs among current smokers.

**Results:**

One thousand one hundred four current smokers included 914 males (82.79%), with an average age of 43.61 years. 58.06% of current smokers reported smoking cessation intention due to GWLs. Logistic regression indicated a higher percentage of smoking cessation intention due to GWLs was among female smokers [OR = 2.41, 95% CI (1.61–3.59)], smokers with smoking intensity < 20 cigarette/day [OR = 1.92, 95% CI (1.44–2.55)], smokers with tobacco burden < 20% [OR = 1.94, 95% CI (1.35–2.79)], and among smokers had plan to quit in a year [OR = 6.58, 95% CI (4.71–9.18). Smokers with higher individual monthly income had lower percentage of smoking cessation intention (OR were 0.35, 0.46 and 0.41). Meanwhile, among 642 current smokers without plan to quit in a year, approximately 40% of them reported smoking cessation intention due to GWLs.

**Conclusions:**

Smoking cessation intention due to the assumed GWLs on cigarette packs is high among current smokers in Shanghai, especially in female smokers, smokers with light tobacco burden and mild nicotine dependence. Incorporating smoking intensity as well as smoking burden into the implementation of GWLs as tobacco control measures would discourage smoking in China.

## Background

Tobacco consumption is the leading cause of death in the world, and frequent inhalation of cigarette smoke toxicants causes both short-term and long-term health effects [1, 2]. Surgeon General’s Advisory Committee (SGAC) of United States indicates tobacco smoking is a health hazard of sufficient importance, and is associated with the increased risk of health problems [3]. Generally, tobacco smoking has negative health effect and is the single largest preventable cause of morbidity and mortality all over the world [4]. World Health Organization (WHO) estimates that the number of people addict to tobacco smoking is over 1 billion, and over 8 million people will die from tobacco related diseases by 2030 if the current trend continues [5]. In order to curb the elevated prevalence of tobacco smoking, many public health interventions have been attempted in efforts, including health literacy campaigns, targeted smoking bans, advertise restrictions, graphic warning labels and excise taxes, etc. [6, 7].

As a way of communication with consumers, cigarette package has become more important following restrictions on other types of tobacco advertisement [8], and is a key component of marketing mix for tobacco industries that aimed at reinforcing brand appeal and discouraging negative cognition which might cause smokers quitting [9]. Overwhelming studies demonstrate that advertising exposure predicts increased trend of tobacco smoking behavior globally [10, 11]. A systematic review indicates the causal relationship between advertisement exposure and youth smoking initiation and adult smoking continuation, and tobacco advertisement also contributes to higher rates of smoking initiation and smoking cessation relapse [10]. The WHO Framework Convention on Tobacco Control (FCTC) recommends the removal of all tobacco industry branding and mandates graphic warning labels on cigarette package, which is crucial for decreasing the high prevalence of tobacco consumption worldwide [12].

In recent years, tobacco control advocates have sought to place health information on cigarette package [8]. Based on communication and health behavior theories [13], message impact framework has been established and applied successfully in cigarette health warning labels (HWLs) research [14]. The message impact framework assumes that features of HWLs will influence smokers’ behavior by a chain of psychological events which ultimately changes their smoking behaviors [15]. Graphic warning labels (GWLs), as a kind of HWLs on tobacco product package, communicate health risks associated with tobacco use and serve as a population level smoking cessation measure [16]. GWLs have been proven to be effective in eliciting negative responses to tobacco smoking, increasing reported intention to quit, and modifying beliefs about tobacco smoking dangers [17, 18]. A study in Shanghai demonstrated that approximately 80% of smokers would consider smoking less, and 48% of smokers reported the intention to quit smoking due to the assumed GWLs on cigarette package [19], and another study in California and North Carolina indicated that 40% of current smokers attempted to quit smoking due to pictorial warning labels [15]. Warning labels with larger graphics in addition to textual messages are associated with a greater impact than smaller text only labels. Observational as well as randomized controlled trails indicate increased tobacco smoking cessation in population where GWLs have been implemented [16]. The guidelines for placing GWLs on cigarette package were issued in the Article 11 of FCTC by WHO, calls for countries to adopt pictorial labels on cigarette packages, with warnings covering at least 50% in the front and back of the display areas [1]. Even though GWLs have been adopted in many countries, the implementation of GWLs is still under debate in China [17].

China is facing a public crisis with an estimated of 320 million people are smokers. The Global Adult Survey (GATS) conducted in 2010 indicate that nearly 1 million smokers die from tobacco related diseases, and about 52% of non-smokers are exposed to secondhand tobacco smoke [13, 18, 19]. China signed the WHO FCTC in 2003 and implemented the FCTC in 2006, but official assessment report indicates that China has made limited progress toward tobacco control and the current smoking prevalence is still high [13]. To reduce the health burden of tobacco smoking, smokers in China need to be encouraged to quit smoking [20]. However, research conducted in 1996, 2002 and 2010 in China suggests that the majority of current smokers have no intention to quit smoking [20, 21]. So further studies examining intention to quit smoking among smokers and exploring the salient tobacco control policies are crucial to decrease smoking prevalence and reduce diseases burden in China.

In this paper, we conduct a cross-sectional study in rural areas of Shanghai, China. We aim to understand the smoking intensity, smoking duration and smoking cessation intention among current smokers, and to explore how smoking cessation intention will be influenced by the assumed graphic warming labels among smokers in Shanghai, China.

## Methods

### Study population

**Fig. 1:**
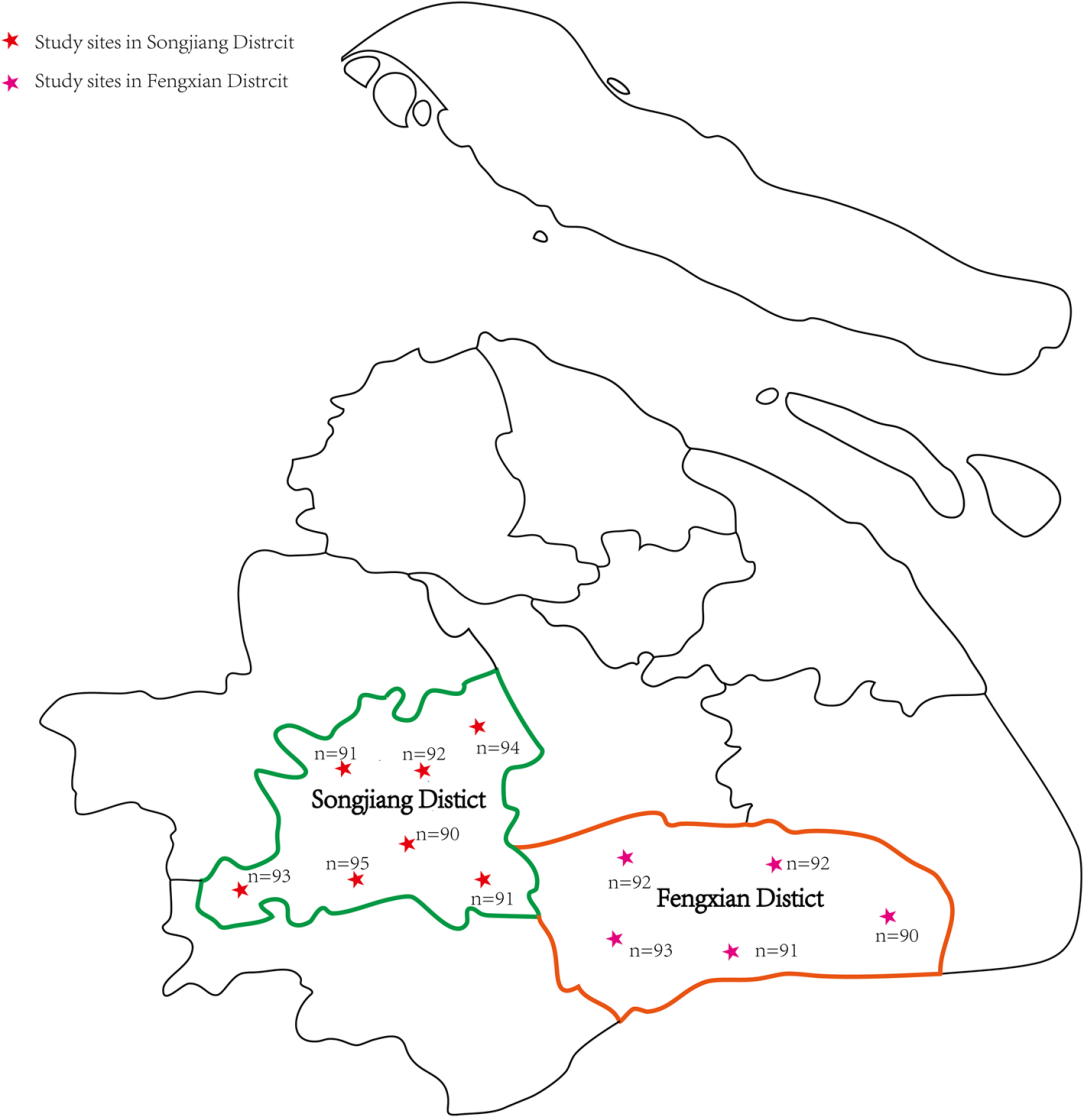
The geographical location of 12 study sites in Songjiang District and Fengxian District, Shanghai

This cross-sectional study was implemented in Songjiang district and Fengxian district of Shanghai during January to June 2021 (Fig. 1). Current smokers in this study did not constitute a random sample of the entire tobacco smoking population in Shanghai, but were selected based on the geographical representation judiciously. In this study, a multistage sampling design was employed to recruit current smokers among the 15 sub-districts of Songjiang district and the 12 sub-districts of Fengxian district, Shanghai. Firstly, seven and five sub-districts were randomly selected from the 15 sub-districts in Songjiang and 12 sub-districts in Fengxian, respectively. Secondly, two residential blocks were selected randomly within each of the selected sub-districts. Thirdly, within each selected residential block, a complete list of home addresses of all households was compiled previously, and then a sample of 100 households were extracted randomly from the list without replacement. Finally, the enumerated 100 households were randomly ordered, and smokers were then approached following the randomized order until 50 current smokers in each selected residential block were surveyed. This study was approved by the Review Board of Shanghai Skin Diseases Hospital affiliated to Tongji University (No. SSDH-21-004 s), and an informed consent paper was signed by each participant before the questionnaire interview. One thousand one hundred four smokers in total (a response rate was 92.00% (1104/1200)) completed the interview and were finally included in data analysis.

### Data collection

**Fig. 2:**
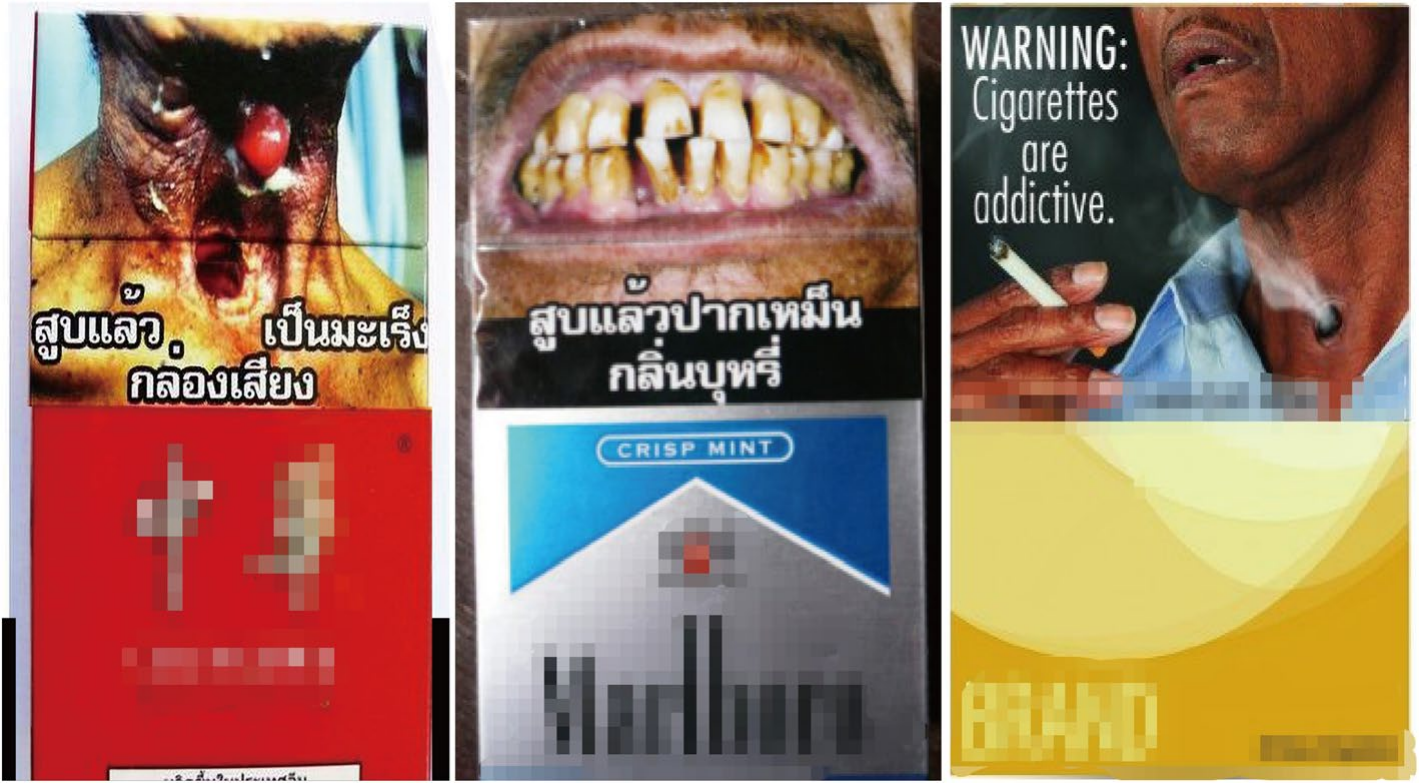
The graphic warning labels (GWLs) on cigarette package used in the investigation

In this study, we employed an electronic questionnaire for data collection. The electronic questionnaire was a Android pad assisted software which was convenient for paperless data input. A pilot study indicated that the split-half reliability coefficient of the electronic questionnaire was 0.87 and the content validity coefficient was 0.85. The electronic questionnaire included (1) demographic information (age, sex, education level, marital status and occupation, etc), (2) history of non-communicable diseases (hypertension, diabetic mellitus, coronary disease, asthma, chronic obstructive pulmonary diseases (COPD), chronic bronchitis, cerebral apoplexy, and cancer), (3) tobacco consumption information (daily tobacco smoking consumption, number of years as a smoker, retail price of daily consumed tobacco, previous tobacco smoking quitting history, etc), and (4) tobacco smoking cessation information (plan to quit smoking in a year, the smoking cessation intention among smokers due to assumed graphic warning labels on cigarette package. The graphic warning labels on cigarette package selected in this study was previously proved as effective GWLs for smoking behavior change [1, 15] (Fig. 2). And we applied face to face interviews to collect the information among current smokers by applying the electronic questionnaire.

### Definition and index calculation

In this study, we define a current smoker as a person who smoked at least 100 cigarettes in a lifetime and still smoke at least once a week at the time of investigation [8, 22], and the second hand tobacco smoke exposure as those who are exposed to tobacco smoke for at least 15 min each day and at least 1 day each week. We define smoking cessation attempt as smokers with quitting attempt for at least 24 h, and we define tobacco smoking relapse as smokers who have made smoking cessation attempt but relapse back to smoking. In this study, we define smoking cessation intention as current smokers who report the intention to quit due to the assumed graphic warning labels on cigarette package. We define smoking duration as the time interval (year) between age at investigation and age at tobacco smoking initiation among current smokers, and then classify it into three groups (‘< 10’ years, ‘10–20’ yeas, and ‘> 20’ years), smoking intensity is defined as the number of cigarettes smoked per day and then categorized as < 20 cigarette/day and ≥ 20 cigarettes/day. Personal tobacco burden is defined as the percentage of individual monthly expense on tobacco purchase divided by individual monthly income, and classified into < 20% and ≥ 20% [22]. In this study, Education is recorded as completed years of schooling and categorized into three categories of 1–9 years (junior high school or lower), 10–12 years (senior high school), and > 12 years (college and above). Individual monthly income is divided into four groups (< 5000 RMB, 5000–10,000 RMB, 10001–20,000 RMB, and over 20,000 RMB).

### Data analysis

We applied SAS software (version 9.3) for data analysis. We described the data as means and standard deviations (SD) for quantitative variables with normal distribution, and as median and interquartile range (IQR) for quantitative variables with skewed distribution. We described the data by frequency counts and proportions (rate) for qualitative variables. Student’s t test, Mann–Whitney U test was applied to examine the group difference between quantitative variables, and Chi-square test was used to examine the group differences between qualitative variables. Logistic regression was applied to calculate the odds ratios (OR) and 95% confidence interval (CI) of smokers who had smoking cessation intention due to the assumed GWLs on cigarette package. Figures were produced to describe the percentage of smokers intended to quit smoking due to GWLs both among smokers with and without the plan to quit in a year. In this study, a *p*-value of less than 0.05 (two-tailed) was considered as statistically significant.

## Results

In this study, 1104 current smokers included 914 males (82.79%), with an average age of 43.61 years (SD was 11.38 years). The majority of current smokers were married (84.96%), with an education of college and above (62.68%). 45.57% of current smokers were official staff or administrative staff. Approximately 74% of smokers had an individual monthly income over 5000 RMB, and 21.83% of them had at least 1 type of non-communicable diseases (NCD). In comparison with female current smokers, male current smokers were elder, had higher proportion of married status, with lower percentage of college and above education, with lower individual monthly income, and with higher prevalence of NCD (Table 1).

**Table 1:** The demographic feature among current smokers by gender in a rural area of Shanghai, China

Variables	Male smokers (*n* = 914)	Female smokers (*n* = 190)	Total smokers (*n* = 1104)
Age (years)^a^, (mean, SD)	44.39 (11.44)	39.80 (10.31)	43.61 (11.38)
Marital status^a^, n (%)			
Married	788 (86.21)	150 (78.95)	938 (84.96)
Unmarried/divorced/widow/widower	126 (13.79)	40 (21.05)	166 (15.04)
Education^a^, n (%)			
Junior High and lower	128 (14.00)	12 (6.32)	140 (12.68)
Senior High	226 (24.73)	46 (24.21)	272 (24.64)
College and above	560 (61.27)	132 (69.47)	692 (62.68)
Occupation^a^, n (%)			
Worker/free occupation/service staff	304 (33.26)	55 (28.95)	359 (32.52)
Official staff/administrative staff	423 (46.28)	79 (41.58)	502 (45.47)
Unemployed/retired/others	187 (20.46)	56 (29.47)	243 (22.01)
Individual monthly income (RMB)^a^, n (%)			
Less than 5, 000	215 (23.52)	64 (33.68)	279 (25.27)
5, 000–10, 000	345 (37.75)	67 (35.26)	412 (32.37)
10, 001–20, 000	245 (26.81)	48 (25.26)	293 (26.54)
Over 20, 000	109 (11.93)	11 (5.79)	120 (10.87)
Residency status, n (%)			
Local resident	784 (85.78)	158 (83.16)	942 (85.33)
Non-local resident	130 (14.22)	32 (16.84)	167 (14.67)
Non-communicable diseases^a^, n (%)			
At least 1 type	228 (24.95)	13 (6.84)	241 (21.83)
0 type	686 (75.05)	177 (93.16)	863 (78.17)

*SD* standard deviation

^a^: the differences between male smokers and female smokers on demographic feature and prevalence of NCD was statistically significant (*P* < 0.05)

### Tobacco smoking condition among current smokers

In this study, the average age of tobacco smoking initiation and the average value of tobacco smoking duration among current smokers were 21.18 years and 22.43 years, respectively. One thousand one hundred four current smokers consumed 15 cigarettes per day on average. The prevalence of smoking intensity < 20 cigarette/day was 60.51%, and the prevalence of personal tobacco burden < 20% was 76.09%. 47.92% of current smokers had attempted smoking cessation previously but all relapsed. The percentage of current smokers who reported second-hand smoke exposure and had plan to quit smoking in a year were 77.63 and 41.85%, respectively. Meanwhile, male current smokers had longer years of tobacco smoking, had lower personal tobacco burden, had higher percentage of smoking cessation attempts and higher percentage of second-hand tobacco smoke exposure, the differences were all statistically significant (*p* < 0.05) (Table 2).

**Table 2:** The smoking intensity, smoking duration, tobacco expenses, and smoking cessation attempt among current smokers in rural area of Shanghai, China

Variables	Male smokers (*n* = 914)	Female smokers (*n* = 190)	Total smokers (*n* = 1104)
Age of tobacco smoke initiation ^a^, (mean, SD)	21.62 (5.17)	19.03 (2.25)	21.18 (4.89)
Years of tobacco smoking^a^, (mean, SD)	22.77 (11.64)	20.77 (10.73)	22.43 (11.51)
Smoking duration (years)^a^, n (%)			
Less than 10	132 (14.44)	34 (17.89)	166 (15.04)
10–20	227 (24.84)	61 (32.11)	288 (26.09)
Over 20	555 (60.72)	95 (50.00)	650 (58.88)
Daily consumed cigarettes on average, (median, IQR)	15 (10–20)	15 (8–20)	15 (10–20)
Smoking intensity (cigarettes/day), n (%)			
Less than 20	554 (60.61)	114 (60.00)	668 (60.51)
Equal or over 20	360 (39.39)	76 (40.00)	436 (39.49)
Tobacco retail price usually purchased, (RMB), (median, IQR)	23 (18–40)	20 (19–40)	23 (15–40)
Monthly expense on tobacco purchase (RMB) ^a^, (median, IQR)	600 (300–1000)	1000 (1000–1000)	700 (300–1000)
Personal tobacco burden ^a^, n (%)			
Less than 20%	734 (80.31)	106 (55.79)	840 (76.09)
Equal or over 20%	180 (19.69)	84 (44.21)	264 (23.91)
Previous smoking cessation attempt ^a^, n (%)			
Yes	499 (54.60)	30 (15.79)	529 (47.92)
No	415 (45.40)	160 (84.21)	575 (52.08)
Times for smoking cessation attempt, (median, IQR)	2 (1–3)	2 (2–7)	2 (1–3)
The longest days of smoking cessation, (median, IQR)	30 (10–150)	30 (30–60)	30 (10–150)
Second hand smoke exposure ^a^, n (%)			
Yes	733 (80.20)	124 (65.26)	857 (77.63)
No	181 (19.80)	66 (34.74)	247 (22.37)
Planned to quit smoking in a year, n(%)			
Yes	414 (45.30)	48 (25.26)	462 (41.85)
No	500 (54.70)	142 (74.74)	642 (58.15)
Smoking cessation intention due to GWLs, n(%)			
Yes	518 (56.67)	123 (64.74)	641 (58.06)
No	432 (43.33)	67 (35.26)	463 (41.94)

*IQR* Interquartile Range, *SD* standard deviation

^a^: the differences between male and female current smokers was statistically significant (*P* < 0.05)

### Smoking cessation intention due to graphic warning labels among current smokers

In this study, 58.06% (641/1104) of current smokers reported smoking cessation intention due to the assumed graphic warning labels on cigarette package. Uni-variable logistic regression in Table 3 indicated that female smokers, smokers with lower monthly income, lower education, lower smoking intensity and lower personal tobacco burden had higher percentage of smoking cessation intention due to GWLs. Meanwhile, smokers with smoking cessation attempt, with plans to quit smoking in a year also had higher percentage of smoking cessation intention due to GWLs. Based on variable selection criteria of logistic regression, variables with a *p* value less than 0.05 in uni-variable logistic regression were selected and fitted into multi-variable logistic regression. Analysis results demonstrated that female smokers [Odd Ratio (OR = 2.41), 95% Confidence Interval (CI) 1.61–3.59] had higher smoking cessation intention than male smokers, smokers with higher individual monthly income had lower percentage of smoking cessation intention (OR were 0.35, 0.46 and 0.41). Smokers with smoking intensity < 20 cigarette/day [OR = 1.92, 95% CI (1.44–2.55)], with tobacco burden < 20% [OR = 1.94, 95% CI (1.35–2.79)] had higher smoking cessation intention due to GWLs on cigarette package. Smokers with plan to quit in a year also had higher smoking cessation intention due to GWLs [OR = 6.58, 95% CI (4.71–9.18)] (Table 3).

**Table 3:** The percentage of smoking cessation intention due to graphic warning label on cigarette package among current smoking in rural area of Shanghai, China

Variables	Percentage of smoking cessation intention	Uni-variate Logistic Regression		Multi-variate Logistic Regression	
		OR	95% CI	OR	95% CI
Age (years)	–	1.00	0.99–1.02	1.01	0.99–1.02
Sex, n (%)					
Female	123 (64.74)	***1.40***	***1.02–1.94***	***2.41***	***1.61–3.59***
Male	518 (56.67)	1.00	–	1.00	–
Education, n (%)					
Junior High or lower	90 (64.29)	1.00	–	1.00	–
Senior High	177 (65.07)	1.04	0.68–1.59	1.49	0.85–2.62
College and above	374 (54.05)	***0.65***	***0.45–0.95***	0.75	0.44–1.28
Individual monthly income (RMB), n (%)					
Less than 5, 000	192 (68.82)	1.00	–	1.00	–
5, 000–10, 000	212 (51.46)	***0.48***	***0.35–0.66***	***0.35***	***0.23–0.53***
10, 001–20, 000	168 (57.34)	***0.61***	***0.43–0.86***	***0.46***	***0.29–0.73***
Over 20, 000	69 (57.50)	***0.62***	***0.39–0.95***	***0.41***	***0.24–0.72***
Non-communicable diseases, n (%)					
At least 1 type	145 (60.17)	1.12	0.84–1.50	1.17	0.82–1.67
0 type	496 (57.47)	1.00	–	1.00	–
Smoking intensity (cigarettes/day), n (%)					
Less than 20	425 (63.62)	***1.78***	***1.39–2.28***	***1.92***	***1.44–2.55***
Equal or over 20	216 (49.54)	1.00	–	1.00	–
Personal tobacco burden, n (%)					
Less than 20%	513 (61.07)	**1.67**	**1.26–2.20**	***1.94***	***1.35–2.79***
Equal or over 20%	128 (48.48)	1.00	–	1.00	–
Previous smoking cessation attempt, n (%)					
Yes	337 (63.71)	***1.57***	***1.23–1.99***	1.30	0.94–1.80
No	304 (52.87)	1.00	–	1.00	–
Planned to quit smoking in a year, n(%)					
Yes	368 (79.65)	***5.29***	***4.02–7.97***	***6.58***	***4.71–9.18***
No	273 (42.45)	1.00	–		
Smoking duration (years), n (%)					
Less than 10	94 (56.63)	1.00	–	–	–
10–20	158 (54.86)	0.93	0.63–1.37	–	–
Over 20	389 (59.85)	1.14	0.81–1.61	–	–
Marital status, n (%)					
Married	544 (58.00)	0.98	0.70–1.37	–	–
Divorced/widow/widower/unmarried	97 (58.43)	1.00	–	–	–
Secondhand smoke exposure, n(%)					
Yes	491 (57.29)	0.87	0.65–1.16	–	–
No	150 (60.73)	1.00	–	–	–

Multi-variate logistic regression for intention to quit smoking due to graphical warning label on cigarette package

*OR* Odds Ratio, *CI* Confidence Interval

### Effect of graphic warning labels on smokers’ smoking cessation intention

**Fig. 3:**
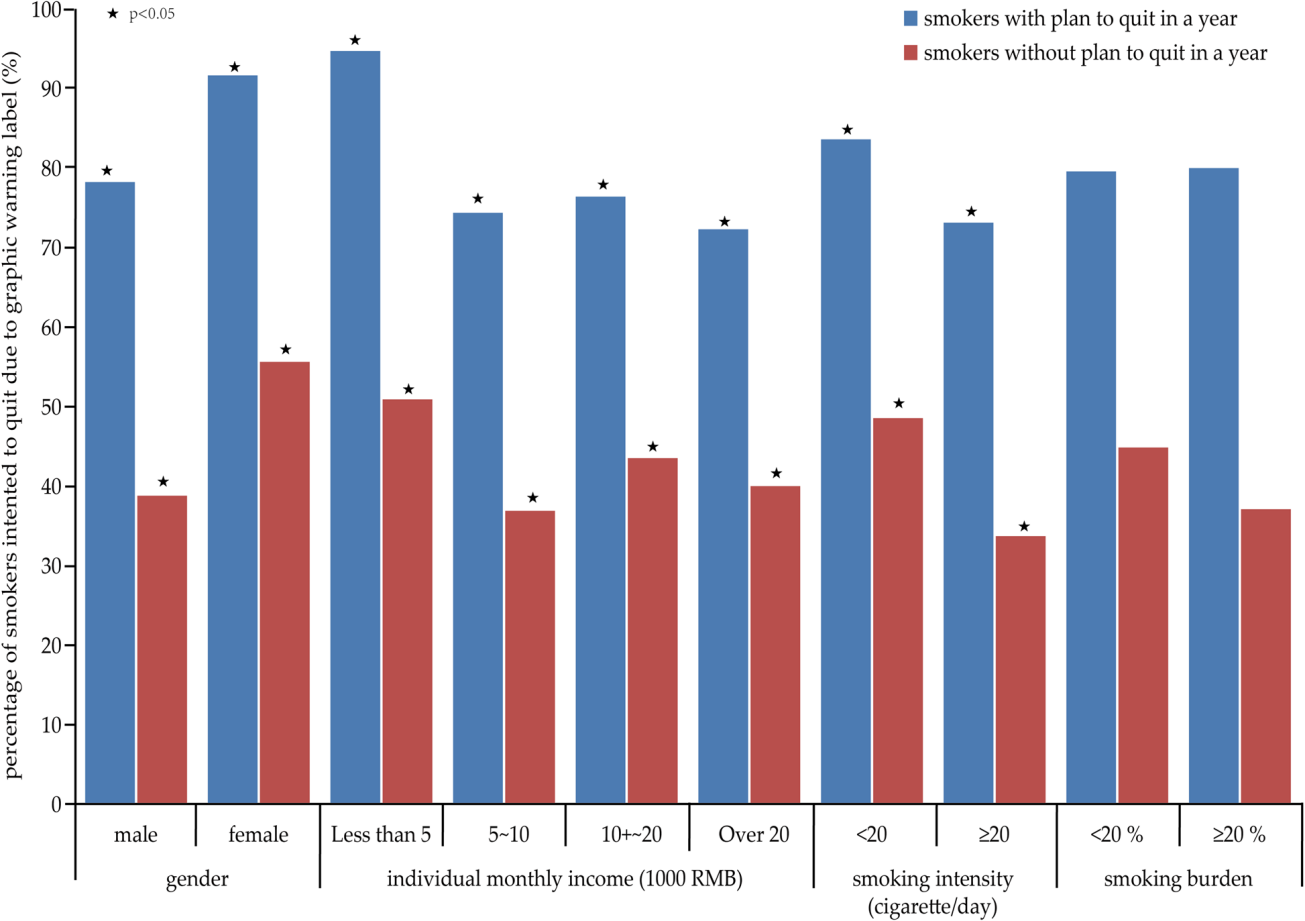
The percentage of smoking cessation intention due to graphic warning labels among smokers with and without plan to quit in a year

In this study, 41.85% of current smokers had plan to quit in a year, and over 58% of current smokers reported smoking cessation intention due to GWLs. In order to evaluate the exclusive effect of GWLs on smokers’ intention to quit, we divided 1104 current smokers into smokers with plan to quit (Group A, *n* = 462) and smokers without plan to quit (Group B, *n* = 642). Figure 3 showed that over 75% of current smokers in Group A and approximately 40% of current smokers in Group B had smoking cessation intention due to GWLs, in spite of their gender, individual monthly income, tobacco smoking intensity and smoking burden. Meanwhile, female smokers (χ^2^_(group-A)_ = 4.77, χ^2^_(group-B)_ = 12.82, *P* < 0.05), smokers with lower monthly income(χ^2^_(group-A)_ = 21.71, χ^2^_(group-B)_ = 8.22, *P* < 0.05), with smoking intensity < 20 cigarettes/day (χ^2^_(group-A)_ = 7.37, χ^2^_(group-B)_ = 13.96, *P* < 0.05) had higher percentage of smoking cessation intention due to GWLs both among current tobacco smokers with and without plan to quit (Fig. 3).

## Discussion

In this study, we identified that over 58% of current smokers had smoking cessation intention due to GWLs on cigarette package. Moreover, about 40% of current smokers who had no plan to quit in a year reported smoking cessation intention due to GWLs. The findings were in line with previous studies [8, 23]. Brewer, et al. [24] demonstrated that affixing GWLs on cigarette package encouraged about 40% of 2149 current smokers planned to quit, which was consistent with findings in this study. GWLs increased smoking cessation intention by increasing attention to the physical harm warnings, inducing emotional response, eliciting negative reactions, avoidance of warning and considering the warning message. In light of the sufficient evidence of GWLs for tobacco control, the fraction of countries having GWLs increased significantly from 15% in 2007, to 55% in 2014 and to over 61% in 2017 [13].

Anh Ngo, et al. [12] demonstrated that GWLs were associated with a 0.9–3 percentage point decrease in adult smoking prevalence and with a reduction of 230–287 sticks of per capita cigarette consumption. In this study, we noticed that 41.85% of smokers in Shanghai had plan to quit smoking in a year, which was higher than that in ITC China Survey in 2006 (26%), and Shanghai Tobacco Control Survey in 2013 (20%) [20]. Meanwhile, more current smokers would consider to quit smoking due to GWLs, so we predicted that the prevalence of tobacco smoking would decrease gradually if GWLs was adopted in Shanghai, China. Whereas, we should notice that 47.92% of current smokers in this study had previously tried to quit smoking but all relapsed. The high percentage of smoking cessation intention were not equal to the high percentage of actual cessation behavior in the future, so incorporating tobacco control measures covering the repeated intervention and professional counseling contrapuntally would promote the smoking cessation intention transferring into actual cessation behavior.

Previous studies proved that smokers with heavier nicotine dependence were not fully process graphic health warning labels, and graphic warning labels might not change their demand for cigarette, or strongly influence their motivation to quit [25, 26]. In this study, we identified that current smokers with lower smoking intensity, longer smoking duration, and lower tobacco burden had higher smoking cessation intention, which was in line with findings of previous studies. Reasons for GWLs induce smokers consider quitting smoking are well explained by the message impact framework [14, 15], some studies also suggest that GWLs may prompt defensive reactions in smokers that cause warnings to have effects that are opposite of what is intended [27]. In China, a long smoking duration predicts the elder age and lower monthly income, smokers with longer smoking duration are also prone to have chronic diseases problems, makes them sensitive to tobacco control measures including GWLs on cigarette package. Meanwhile, lower smoking intensity indicates a mild nicotine dependence among smokers, so they are prone to change their smoking habits with the assumed GWLs on cigarette package.

A growing body of researches have demonstrated that women experience significant health disparities related to tobacco smoke [28, 29]. Women have lower rates of successfully quitting, and experience greater risk of certain health consequences of smoking [30]. Furthermore, women smokers response more strongly to GWLs on cigarette package, and are more likely to consider smoking cessation due to GWLs [31, 32]. In this study, we identified that female smokers had over 2.4 folds of smoking cessation intention due to GWLs in comparison with male smokers, which was in line with previous evidences that graphic warning labels were more effective in motivating female smokers to quit [32]. This might attribute to the fact that women are more sensitive to malignancies and respiratory illnesses depicted on top of the cigarette package, and then induced strong negative emotions [4, 31, 32]. We recommend that sex differences in smoking cessation intention due to GWLs on cigarette packs should be considered when adopting GWLs into tobacco control measures.

To our knowledge, this study is the first attempt to exclusively estimate how smoking cessation intention would be influenced by the assumed graphic warning labels (GWLs) in Shanghai, China. The findings in this study indicate that smoking cessation intention due to the assumed GWLs on cigarette packs is high among current smokers, so incorporating GWLs into tobacco control measures would discourage tobacco smoking in Shanghai, China. A key strength of this study was that data was collected through face to face interview by an Android pad assisted electronic questionnaires, the automated logical check schedule in data collection process and the whole course audio record ensured a high data quality. In this study, we applied a special multistage sampling methods due to the low prevalence of tobacco smoking among female population in China (about 3%), in which we choose one male smoker and one female smoker from each selected households whenever possible to increase the sample size of female smoker, is another strengthen of this study.

There are some limitations in this study. Firstly, the sample of smokers was judiciously selected based on the geographical representation, but not a random sample of the entire smoking population in Shanghai, and the 1104 smokers in this study was a relatively smaller sample in comparison with the large number of tobacco smokers in Shanghai, which limited the generalization of findings in this study. Secondly, smoking cessation intention due to the assumed GWLs on cigarette package was just the attitude of smokers but not the actual behavior change, which impeded the observation of real effect, so implementing an intervention and follow-up study would be a major step forward to evaluate the actual effect of GWLs as tobacco control measure. Thirdly, we only employed graphic warning labels to explore its association with smoking cessation intention among smokers, however, other factors including physicians’ advice for quit, tobacco retail price increase, tobacco control advertisements as well as tobacco control campaigns such as ‘the Chinese International Quit and Win Competition’ may also affect the smoking cessation intention among current smokers. Fourth, answers were self-reported by participants which might induce recall bias and report bias, and other confounding factors such as experienced tobacco smokers, nicotine dependence and exposure time duration to GWLs could also bias the results. So the incorporation of some improvements should be considered in further studies.

## Conclusions

Smoking cessation intention due to the assumed GWLs on cigarette packs is high among current smokers in Shanghai, especially among female smokers, smokers with light tobacco burden and mild nicotine dependence. Incorporating smoking intensity as well as smoking burden into the implementation of GWLs as tobacco control measures would discourage smoking in China.

## Data Availability

Data in this study can be made available upon request to the corresponding author.

## Abbreviations

SGAC: Surgeon General’s Advisory Committee
WHO: World Health Organization
GATS: Global Adult Tobacco Survey
FCTC: WHO Framework Convention on Tobacco Control
HWLs: Health warning labels
COPD: Chronic Obstructive Pulmonary Disease
OR: Odds Ratio
CI: Confidence Interval
DAGs: Directed Acyclic Graphs
SD: Standardized Deviation
NCD: Non-communicable Diseases
IQR: Interquartile Range
ITC: International Tobacco Control
